# Sequence, distribution and chromosomal context of class I and class II pilin genes of *Neisseria meningitidis* identified in whole genome sequences

**DOI:** 10.1186/1471-2164-15-253

**Published:** 2014-04-01

**Authors:** Mirka E Wörmann, Corey L Horien, Julia S Bennett, Keith A Jolley, Martin C J Maiden, Christoph M Tang, Ellen L Aho, Rachel M Exley

**Affiliations:** 1 Sir William Dunn School of Pathology, University of Oxford, Oxford OX1 3RE, UK; 2 Department of Biology, Concordia College, Moorhead, MN, USA; 3 Department of Zoology, University of Oxford, Oxford OX1 3PS, UK

**Keywords:** Type four pilus, *Neisseria meningitidis*, Class I pilin, Class II pilin, Antigenic variation

## Abstract

**Background:**

*Neisseria meningitidis* expresses type four pili (Tfp) which are important for colonisation and virulence. Tfp have been considered as one of the most variable structures on the bacterial surface due to high frequency gene conversion, resulting in amino acid sequence variation of the major pilin subunit (PilE). Meningococci express either a class I or a class II *pilE* gene and recent work has indicated that class II pilins do not undergo antigenic variation, as class II *pilE* genes encode conserved pilin subunits. The purpose of this work was to use whole genome sequences to further investigate the frequency and variability of the class II *pilE* genes in meningococcal isolate collections.

**Results:**

We analysed over 600 publically available whole genome sequences of *N. meningitidis* isolates to determine the sequence and genomic organization of *pilE*. We confirmed that meningococcal strains belonging to a limited number of clonal complexes (ccs, namely cc1, cc5, cc8, cc11 and cc174) harbour a class II *pilE* gene which is conserved in terms of sequence and chromosomal context. We also identified *pilS* cassettes in all isolates with class II *pilE*, however, our analysis indicates that these do not serve as donor sequences for *pilE/pilS* recombination. Furthermore, our work reveals that the class II *pilE* locus lacks the DNA sequence motifs that enable (G4) or enhance (Sma/Cla repeat) pilin antigenic variation. Finally, through analysis of pilin genes in commensal *Neisseria* species we found that meningococcal class II *pilE* genes are closely related to *pilE* from *Neisseria lactamica* and *Neisseria polysaccharea,* suggesting horizontal transfer among these species.

**Conclusions:**

Class II pilins can be defined by their amino acid sequence and genomic context and are present in meningococcal isolates which have persisted and spread globally. The absence of G4 and Sma/Cla sequences adjacent to the class II *pilE* genes is consistent with the lack of pilin subunit variation in these isolates, although horizontal transfer may generate class II pilin diversity. This study supports the suggestion that high frequency antigenic variation of pilin is not universal in pathogenic *Neisseria*.

## Background

*Neisseria meningitidis*, the meningococcus, is an important human pathogen, being a leading cause of septicaemia and bacterial meningitis. Systemic disease has a high case fatality rate and can progress rapidly over the course of a few hours [1]. Different epidemiological patterns of meningococcal disease are observed across the world and Multi-Locus Sequence Typing (MLST) has been instrumental in characterising strains and identifying major pathogenic lineages (clonal complexes) responsible for disease [2]. Many *N. meningitidis* isolates from invasive disease belong to ‘hyperinvasive lineages’ [3], such as clonal complexes (cc)- 5, cc11, cc269, cc32 and cc41/44. Whole genome sequence (WGS) analyses have recently been carried out on large isolate collections, including those used to develop MLST [2,4]. These WGS data represent a valuable resource for investigating the frequency and variability of genes encoding meningococcal virulence traits.

Type four pili (Tfp) are important virulence factors for *N. meningitidis*, contributing to colonisation and disease [5]. Adhesion of meningococci to epithelial and endothelial cells is mediated by Tfp interacting with host cells, promoting bacterial aggregation and mediating microcolony formation [6]. *Neisseria* Tfp are also essential for the acquisition of exogenous DNA and emergence of strains with novel, heritable characteristics [7]. Along with LPS, porins and the polysaccharide capsule, Tfp have been considered as key meningococcal surface structures which undergo extensive variation. Studies on the Tfp of the related pathogen *Neisseria gonorrhoeae* have revealed that changes in the amino acid sequence of PilE (pilin), the major subunit of Tfp, arise through introduction of segments of non-expressed *pilS* cassettes into the *pilE* expression locus by a unidirectional DNA recombination event known as gene conversion [8,9]. This process is enhanced by the presence of the Sma/Cla repeat and requires the guanine quartet (G4) adjacent to the *pilE* expression locus. The Sma/Cla repeat is a 66 bp element present at the 3′ end of *pilE* that is proposed to bind proteins to facilitate recombination [10]. The G4 sequence is located upstream of *pilE* and is necessary for pilin conversion, putatively by acting as a recombination initiation structure [11,12]. More recently a cis-acting small RNA has been proposed to assist in the melting of the DNA duplex to allow formation of the G4 structure [13]. High frequency antigenic variation generates remarkable PilE diversity, with most of the sequence changes localised to the regions of the protein that are exposed on the surface of the pilus fibre [14], most notably within a region (D-region) located near the C-terminus of the protein. Gonococcal pilin variation has been detected both *in vitro*[15] and in human volunteer studies [16]. Meningococcal pilin variation has also been shown in a limited number of isolates *in vitro*[17] and *in vivo*[18,19] and has been proposed to influence Tfp-mediated adhesion [20-22], serum resistance [23] and to serve as a mechanism for immune evasion.

Two classes of Tfp (class I and class II) have been described in the *Neisseria*, based on the cross reaction of PilE with a monoclonal antibody, SM1 [24]. This antibody recognizes a conserved epitope in class I, but not class II pilin [25]. Furthermore, class II pilins lack most of the D-region typically seen in gonococccal and meningococcal class I pilins [26]. The class II *pilE* gene was initially described in the *N. meningitidis* serogroup C strain, FAM18 and subsequently found in non-pathogenic species, *N. lactamica* and *N. cinerea*[27]. Until recently, all pathogenic *Neisseria* were thought to have the ability to alter the sequence of the major pilin subunit through gene conversion; however, pilin variation was not detected *in vitro* in *N. meningitidis* strain FAM18, or strain NMB, which both express class II pilin [17,28]. Furthermore, in a previous study we found that cc8 and cc11 isolates of *N. meningitidis* collected from the UK over a period spanning 30 years harboured highly conserved class II *pilE* genes which lack variation characteristic of gene conversion [29]. Similar analysis of the sequence of *pilE* genes in meningococcal isolates from China demonstrated that strains in cc1 and cc5 also have a highly conserved class II *pilE* gene [30].

These findings suggest that high frequency *pilE* gene conversion is not universal in pathogenic *Neisseria,* an observation which has important implications for understanding the role of Tfp variation during colonisation, transmission and disease. Therefore, in this study we interrogated genome sequences of over 600 meningococcal isolates to further examine the distribution and sequence conservation of class II *pilE* genes, and to identify differences in the class I and class II *pilE* loci which might explain the lack of variability of the class II pilin gene. Our analyses showed that the class I and class II *pilE* genes are in distinct chromosomal contexts and that isolates with class II *pilE* contain a more limited repertoire of *pilS* cassettes. Furthermore, the G4 and Sma/Cla sequences, which are important for pilin variation, are absent from the class II *pilE* locus. Finally we present evidence of horizontal transfer of class II *pilE* genes between pathogenic and non-pathogenic *Neisseria* species, which has implications for the evolution of these bacteria.

## Results

### Distribution and diversity of class I and class II *pilE* in *N. meningitidis*

Draft whole genome sequences hosted on the PubMLST website [4] were interrogated to determine the distribution and diversity of class II *pilE* genes among *N. meningitidis* isolates. This included the Meningitis Research Foundation Meningococcus Genome Library (MRF-MGL), which contains the sequences of 514 disease-associated isolates from patients in England, Wales and Northern Ireland during a single epidemiological year (2010 to 2011), and which represents recently circulating UK disease causing meningococci. In addition WGS of the 107 strains of *N. meningitidis* used to develop and validate MLST, which comprises a diverse collection of meningococci isolated from 37 countries collected over a 59 year period (1937 to 1996) [2] were examined.

Pilin genes in this collection of isolates were identified by online BLAST analyses using full-length or partial class I or class II *pilE* sequences. Homologous sequences were manually extracted, translated and designated as either class I or class II *pilE* based upon relatedness to known pilin genes and the presence of the SM1 motif (EYYLN) in the translated sequence. It was not possible to identify full-length *pilE* sequences in all WGS, most likely due to incomplete assembly. From the MRF genomes a total of 201 full-length class I *pilE* coding sequences and 31 class II *pilE* coding sequences were identified and in the MLST collection of 107 genomes, 12 full-length class I *pilE* coding sequences and 44 class II *pilE* coding sequences were found (a breakdown of *pilE* analysis is described in Additional file 1). Therefore, in total 213 isolates with a full-length class I *pilE* gene and 75 isolates with a full-length class II *pilE* gene were identified (listed in Additional files 2 and 3). No isolates that contained both a class I and a class II *pilE* gene were identified.

While there was no association of class II *pilE* with serogroup, country or year, the presence of a class II *pilE* gene was consistently associated with a limited group of clonal complexes *i.e*. cc1, cc5, cc8, cc11 and cc174; no WGS data from isolates belonging to these ccs contained a class I *pilE* gene. Only cc4 contained isolates with either class I *pilE* (ID 35 and ID 613) or class II *pilE* (ID 1) (see Additional files 2 and 3). Thus, the data from the 107 MLST and MRF isolate collections confirmed that the presence of class II pilin is a characteristic of a distinct subset of meningococcal lineages.

**Figure 1:**
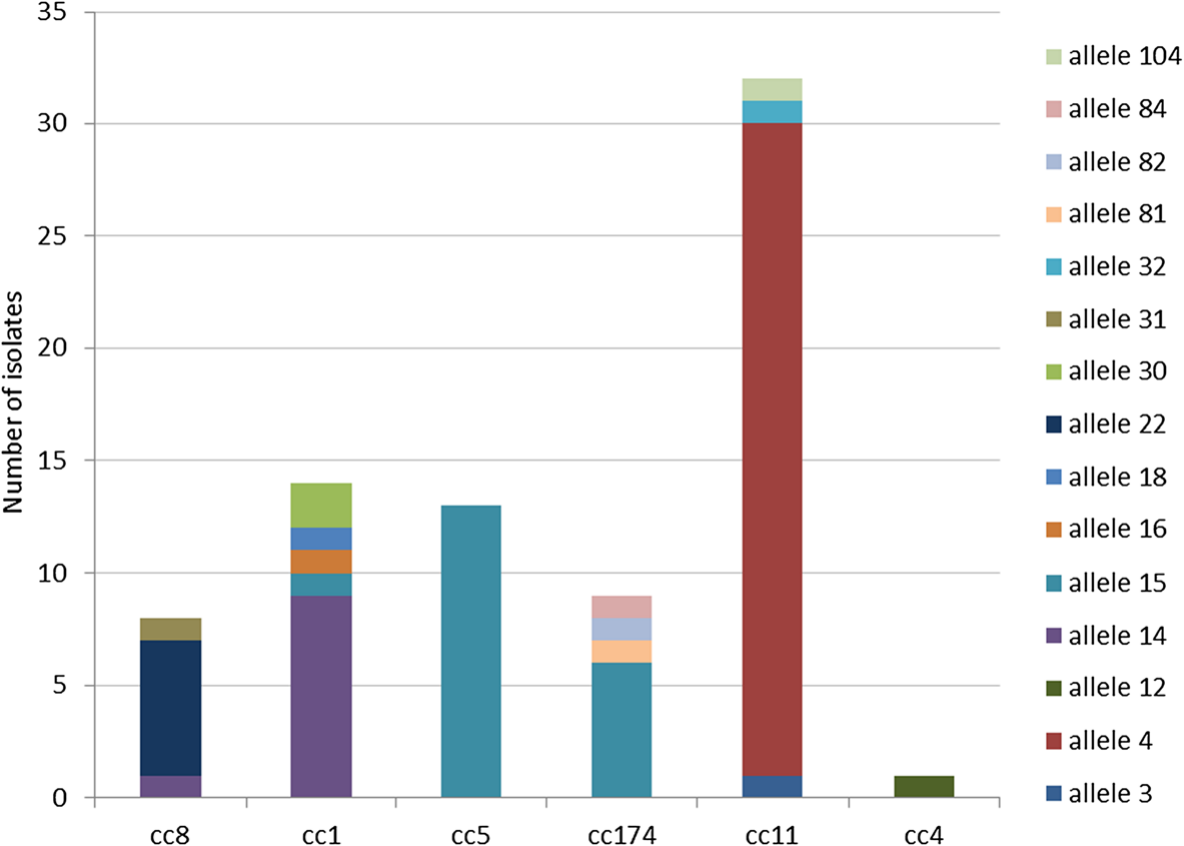
**Distribution of class II *pilE *nucleotide alleles among clonal complexes.** A total of 15 different alleles were identified from 77 meningococcal isolates. Isolates from cc1 displayed the most diversity, with five unique *pilE* sequences found in 14 isolates. All 13 cc5 strains had identical *pilE* sequence. Alleles 14 and 15 were found in isolates from 2 or 3 different clonal complexes respectively. Clonal complex and *pilE* alleles are indicated.

The extent of sequence diversity among the 213 class I and the 75 class II *pilE* sequences identified in this work was examined, including *pilE* sequences from eight available reference genome sequences, providing an additional six class I *pilE* genes and two class II *pilE* sequences (Additional file 4). Allele numbers were assigned to the full-length *pilE* (NEIS0210) coding sequences. Each of the 219 class I *pilE* genes was a unique allele (Table 1), even in strains of the same sequence type, isolated in the same country and year. In contrast, only 15 class II *pilE* alleles were identified from 77 meningococci isolated from 29 countries over 74 years, highlighting the sequence conservation of class II *pilE* genes (Table 1). There was also evidence for an association between certain class II *pilE* alleles and particular clonal complexes (Figure 1). For example, 90% (28/31) of cc11 isolates harboured an identical *pilE* (allele 4). Likewise, all 13 cc5 isolates harboured the same *pilE* coding sequence (allele 15). This allele was also found in strains belonging to cc1 and cc174, suggesting horizontal transfer of class II *pilE*. Further evidence of this was allele 14, which was present in strains belonging to cc1 and cc8.

**Table 1:** **Analysis of class I and class II *pilE *** **sequence diversity**

***pilE***	**No. isolates**	**No. countries**	**Years**	**No. ccs**	**No. alleles**
Class I	219	5	1963-2011	21 + n/a	219
Class II	77	29	1937-2011	6	15

n/a: not assigned to clonal complex.

**Figure 2:**
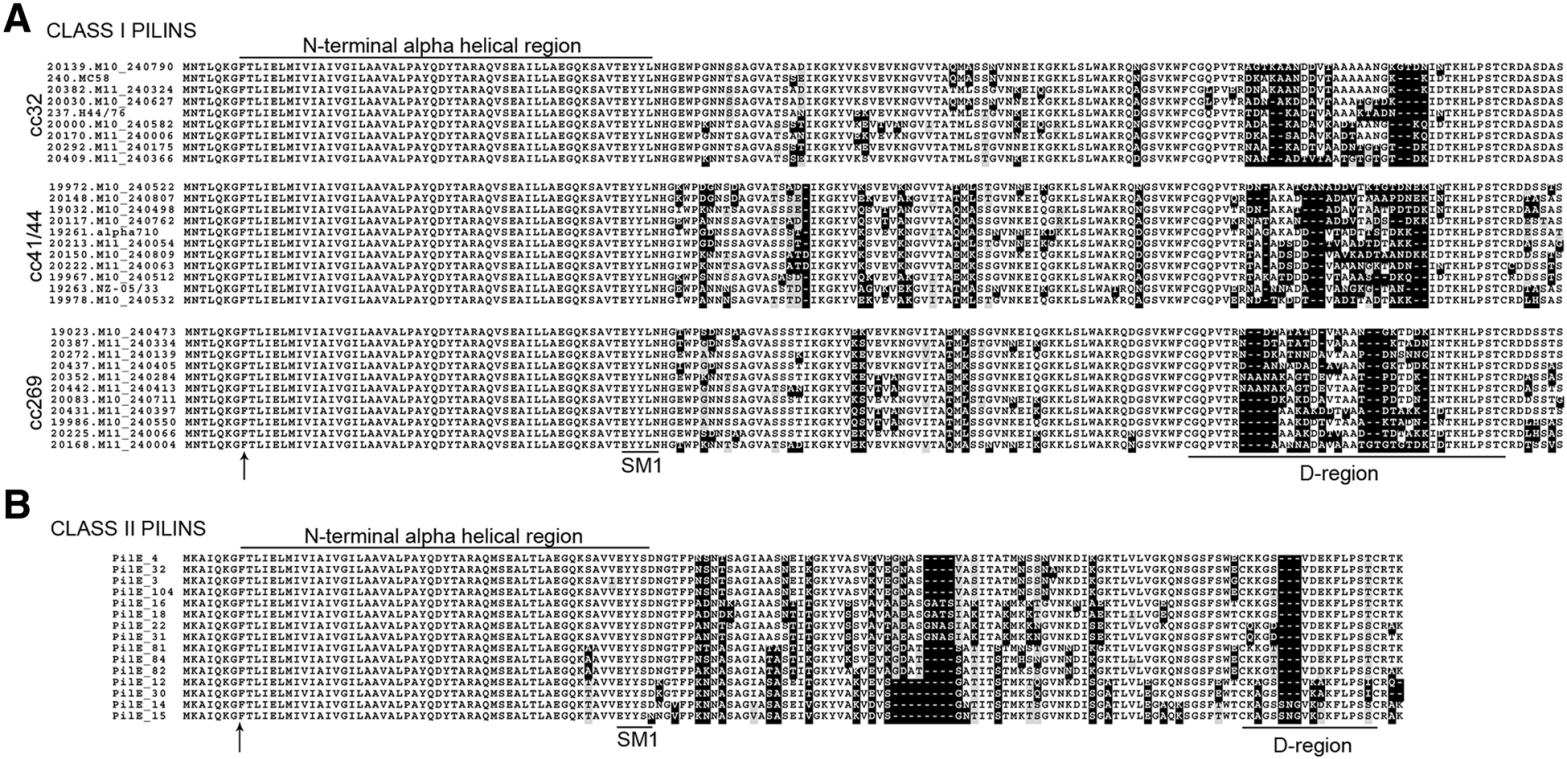
**Clustal W Alignment and Boxshade display of the deduced full-length amino acid sequences of (A) representative class I pilins from cc32, cc41/44 and cc269 and (B) the 15 class II pilin proteins encoded by 77 meningococcal isolates.** For class I pilins, isolate ID and name are indicated. For class II pilins, sequence numbers correspond to the *pilE* allele number assigned by PubMLST. The phenylalanine (F) residue which is the start of mature pilin is shown by an arrow. The SM1 motif at the end of the putative N-terminal α-helical region is indicated. Black background highlights different residues. Grey background text indicates similar residues. The N-terminal region is highly conserved, as for all meningococcal type IV pilins. The D-region (highlighted) is defined by two conserved C-terminal cysteine residues. The hypervariable region of class I pilins is within the D-region. In class II pilins, sequence variation is found over the entire C-terminal domain.

Analysis of the predicted protein sequences showed that for class I pilins most variation occurred within the C-terminal D-region (Figure 2A), as expected based on previous studies [8,14,31]. The 219 different class I *pilE* alleles encode 219 different pilin subunits with 194 different D-regions (not shown). In contrast, alignment of the 15 unique class II pilins we identified demonstrates that variation occurs over the entire C-terminal domain and only six different D-regions are encoded (Figure 2B).

### Analysis of *pilE/pilS* loci in isolates with class I or class II *pilE*

**Figure 3:**
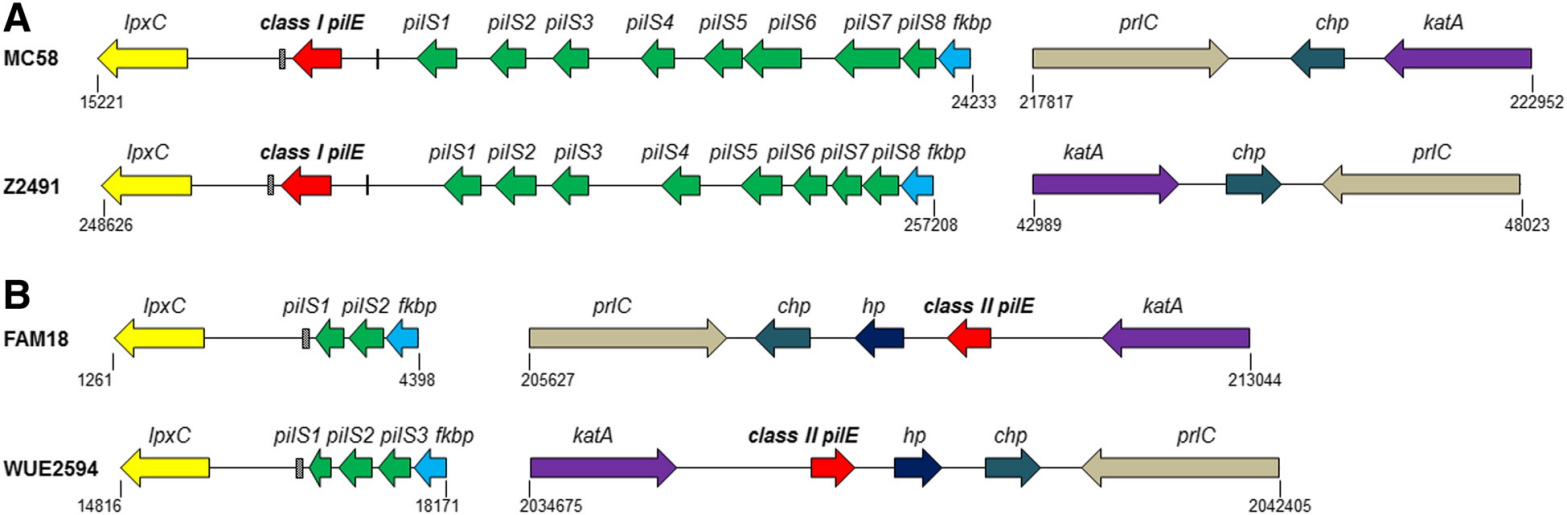
**Schematic diagrams of the positions of *pilE *and *pilS *loci in genomes of reference strains of *N. meningitidis *expressing class I *pilE *(A) or class II *pilE *(B).** Genes and *pilS* cassettes are drawn based on published annotations. The positions on the chromosome are based on NCBI reference sequences: NC_003112.2 (MC58), NC_008767.1 (FAM18), NC_003116.1 (Z2491), and NC_017512.1 (WUE2594). As shown, class II *pilE* genes have a distinct genomic context compared to the class I *pilE* genes. Putative guanine quartet (G4) and Sma/Cla sequences are shown as black vertical line or hatched boxes, respectively. *lpxC*: UDP-3-O-[3-hydroxymyristoyl] N-acetylglucosamine deacetylase), *fkbp*: peptidyl-prolyl cis-trans isomerase, *katA*: catalase, *chp*: conserved hypothetical protein; *hp*: hypothetical protein, *prlC*: putative oligopeptidase A.

In the class I pilin-producing meningococci with closed genome sequences (MC58 [32] and Z2491 [33]) the class I *pilE* gene and eight *pilS* cassettes are found in a single locus, while in the closed genomes of class II pilin-producing meningococci FAM18 [34] and WUE2594 [35], this locus contains a reduced number of *pilS* cassettes and class II *pilE* is located at a distinct locus (Figure 3). The class I *pilE* gene is located between *fkbp* (which encodes a putative peptidyl-prolyl cis-trans isomerase) and *lpxC* (UDP-3-O-[3-hydroxymyristoyl] N-acetylglucosamine deacetylase) (Figure 3A). The silent *pilS* cassettes precede *pilE* and, although there are eight cassettes in each instance, there is variation in their length and spacing. In contrast the class II *pilE* gene is situated between catalase (*katA*) and a hypothetical protein (*hp*), with a conserved hypothetical (*chp*) and putative oligopeptidase A gene (*prlC*) further downstream (Figure 3B). The catalase region in the genomes of MC58 and Z2491 does not contain a class II *pilE* gene (Figure 3A). Finally, in FAM18 and WUE2594, two or three *pilS* cassettes respectively are located in the region between *fkbp* and *lpxC* (Figure 3B).

**Figure 4:**
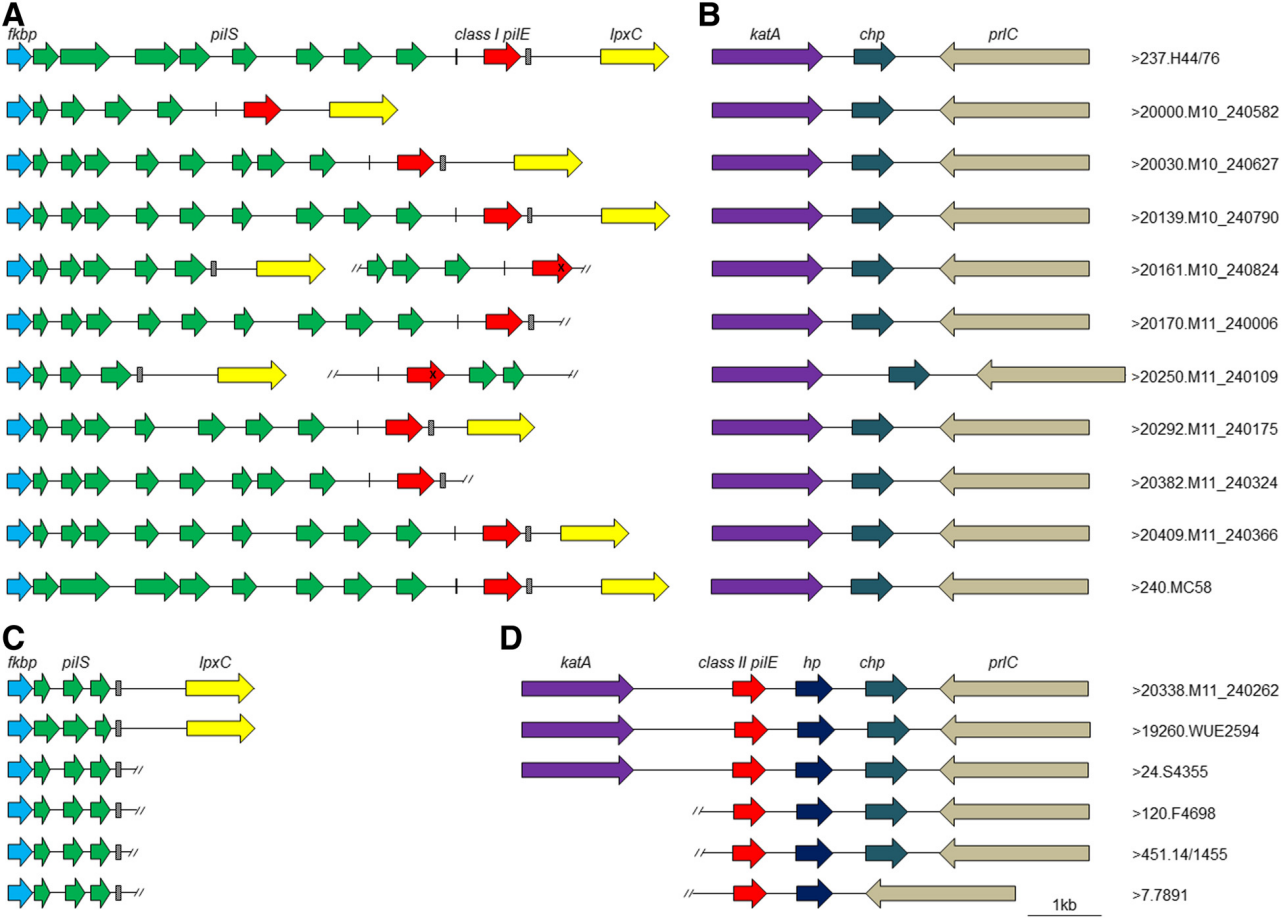
**Schematic diagrams of *fkbp-lpxC *and *katA*-*prlC *regions in a subset of meningococcal isolates with class I *pilE *(A-B) and class II *pilE *genes (C-D). (A)** Open reading frames and *pilS* cassettes in the region flanking the class I *pilE* gene were annotated in isolates belonging to cc32. Class I *pilE* genes were mostly found adjacent to *pilS* cassettes in the *fkbp*-*lpxC* region. **(B)** The *katA*-*prlC* region of cc32 isolates does not contain a class II *pilE* gene. **(C)** Open reading frames and *pilS* cassettes were annotated in the *fkbp*-*lpxC* region of isolates belonging to cc5. These strains have three *pilS* cassettes which are highly conserved. **(D)** The class II *pilE* gene is located next to the catalase gene (*katA*) and is genetically linked to a gene encoding a hypothetical protein (*hp*). The ID and name of each isolate is indicated. Sequence type and provenance of each strain can be found in Additional files 2 and 3. Diagonal lines represent the end of the contig. X indicates the gene encodes an atypical pilin subunit which is unlikely to result in production of functional Tfp. Putative guanine quartet (G4) and Sma/Cla sequences are shown as black vertical line or hatched boxes, respectively. *lpxC*: UDP-3-O-[3-hydroxymyristoyl] N-acetylglucosamine deacetylase), *fkbp*: peptidyl-prolyl cis-trans isomerase, *chp*: conserved hypothetical protein; *prlC*: putative oligopeptidase A. Scale bars represent 1 kb.

To assess the chromosomal context of class I and class II *pilE* genes and their associated *pilS* repertoires in different groups of meningococcal isolates, we annotated the *fkbp*-*lpxC* region and the *katA*-*prlC* regions in a subset of WGS in the PubMLST database. Where possible groups of isolates with the same sequence type or in the same clonal complex were examined, to enable comparisons both within and between groups of related meningococci. The *pilS* cassettes were defined on the basis of their homology to *pilE* coding sequences. In some cases the lack of available contiguous sequence prevented identification of adjacent open reading frames; examples of class I and class II *pilE*/*pilS* regions from genomes where the assembly was sufficient are shown in Figure 4 and Additional files 5 and 6.

Class I *pilE* genes were found adjacent to *pilS* cassettes and between *fkbp* and *lpxC* in 29 of the 34 annotated genomes. In the remaining five genomes, it was not possible to determine whether *pilE* was adjacent to *lpxC* (Isolate IDs 20170 and 20382, Figure 4) or *pilE* genes encoding atypical pilin variants (with an extended and truncated C-terminus, respectively) were found, but these were not within in the *fkbp*-*lpxC* locus (cc32 isolates ID 20161 and ID 20250, Figure 4A). Likewise in cc269 isolate M10_240550 (ID 19986), the *pilE*/*S* locus was interrupted by a region homologous to the *tspB* region of strain FAM18 (see Additional file 5). None of these isolates harboured a *pilE* gene in the *katA*-*prlC* region (Figure 4B).

Analysis of *pilS* cassettes in the *fkbp*-*lpxC* region of the class I *pilE*-containing isolates revealed diversity in the spacing, number and sequence of the silent cassettes, both among clonal complexes and among isolates belonging to the same clonal complex (see Table 2 and schematic loci in Figure 4 and Additional file 5). This diversity was particularly evident in cc41/44, where we identified 52 different *pilS* sequences from a total of 84 cassettes from 12 genomes. It should be noted that the analysis of class I strains was specifically targeted to annotate *pilS* cassettes within the *fkbp*-*lpxC* region and the presence of additional *pilS* cassettes elsewhere in these genomes could not be excluded. Therefore this analysis represents the minimum diversity of *pilS* in isolates with class I *pilE*.

**Table 2:** ***pilS *** **sequence diversity in isolates with class I and class II *pilE***^**1**^

**Clonal complex**	***pilE *** **gene**	**Number of isolates**	***pilS *** **cassettes per isolate**	**Total number of *pilS *** **cassettes**	**Total number of *pilS *** **alleles**
cc1	class II	5	1-3	13	8
cc5	class II	6	3	18	8
cc8	class II	8	3	24	4
cc11	class II	32	2	64	4
cc174	class II	9	2-4	27	6
cc269	class I	10	5-9	82	19
cc41/44	class I	12	5-11	84	52
cc32	class I	9	4-9	70	26

^1^Isolates and *pilS* cassettes included in this analysis are shown in Figure 4, Additional file 5, and Additional file 6. Isolates ID 19986 (cc269), ID 20161 (cc32), and ID 20250 (cc32) were excluded due to incomplete contig assembly in regions containing *pilE/pilS* loci.

The *pilS* and *pilE* sequences within each isolate were examined for evidence of past intra-strain gene conversion events. Of note, for all isolates with a class I *pilE* gene, it was possible to identify *pilS* cassettes with sequences corresponding to those found in the PilE D-region. In most cases the D-region was a hybrid, likely resulting from multiple recombination events, whilst in some instances a single *pilS* donor could be proposed based on full sequence identity (not shown).

Annotation of genomes with class II *pilE* revealed that this gene was present in a region defined as the *katA*-*prlC* region (annotated regions are shown in Figure 4C and D, and Additional file 6). All these regions had identical genetic organisation, except isolate 7891 (ID7, Finland, 1975), which lacks the conserved hypothetical protein (*chp*, NMC0207 in FAM18). Furthermore, all genomes with class II *pilE* had fewer *pilS* cassettes than class I isolates - usually two or three in the region between *fkbp* and *lpxC* (Figure 4C and Table 2). Further BLAST analyses of these class II genomes were performed to identify whether *pilS* cassettes were also in other loci. The only homologous sequences identified were located on contigs containing the region between *fkbp* and *lpxC* (not shown), suggesting that there was only a single *pilS* locus in these isolates. Although incomplete genome assembly preventing additional *pilS* identification in this study cannot be excluded, the fully assembled, closed genome sequences of reference strains FAM18 and WUE2594 do not contain *pilS* cassettes located elsewhere.

In comparison to strains with class I *pilE*, the *pilS* regions in these class II isolates were highly conserved within a clonal complex in terms of number and arrangement of *pilS* cassettes (see Figure 4 and Additional file 6). For example, there were only four different *pilS* sequences out of 64 cassettes in 32 cc11 isolates (Table 2). The *pilS* cassettes in class II isolates shared greater similarities with class I *pilE* than class II *pilE* as previously observed [36]. Furthermore, we were unable to identify any *pilS* cassettes encoding sequences corresponding to those found in the D-regions of any class II pilin proteins. This finding is consistent with the hypothesis that, in these class II isolates, *pilS* cassettes are not functioning as donors for intra-strain recombination events associated with pilin antigenic variation.

### Analysis of DNA sequences associated with *pilE* gene conversion

The G4 and Sma/Cla elements have been shown to be required for efficient antigenic variation of pilin [12,37]*.* Each of these sequences was found in the region 5′ or 3′ to the class I *pilE* gene, respectively (Additional file 7). Four isolates lacked a full length Sma/Cla adjacent to *pilE*, three of which also presented atypical *pilE* sequence and/or arrangement; for example, cc41/44 isolate (ID19266) (Additional file 5), has an atypical *pilE* sequence and lacks a Sma/Cla sequence, while cc32 isolates ID 20161 and ID 20250 (Figure 4) have a Sma/Cla sequence adjacent to *pilS* but in these genomes *pilE* is no longer found in the *fkbp*-*lpxC* region.

We also analysed the regions flanking the class II *pilE* alleles for presence of the Sma/Cla and G4 sequences. We did not detect sequences corresponding to a functional G4 motif (5′-GGGTGGGTTGGGTGGG-3′) [12] in the region 5′ to any class II *pilE* gene. Furthermore, the Sma/Cla repeat was absent from the region 3′ to the class II *pilE* genes, although sequences identical or highly homologous to the published meningococcal Sma/Cla repeat [37] were present in the *pilS* loci of these isolates (see Figure 4 and Additional files 6 and 7). Therefore, the absence of motifs that are important for *pilE*/*pilS* recombination adjacent to the class II *pilE* gene is likely to contribute to the lack of antigenic variation of class II *pilE*.

### Defining meningococcal class II pilins

**Figure 5:**
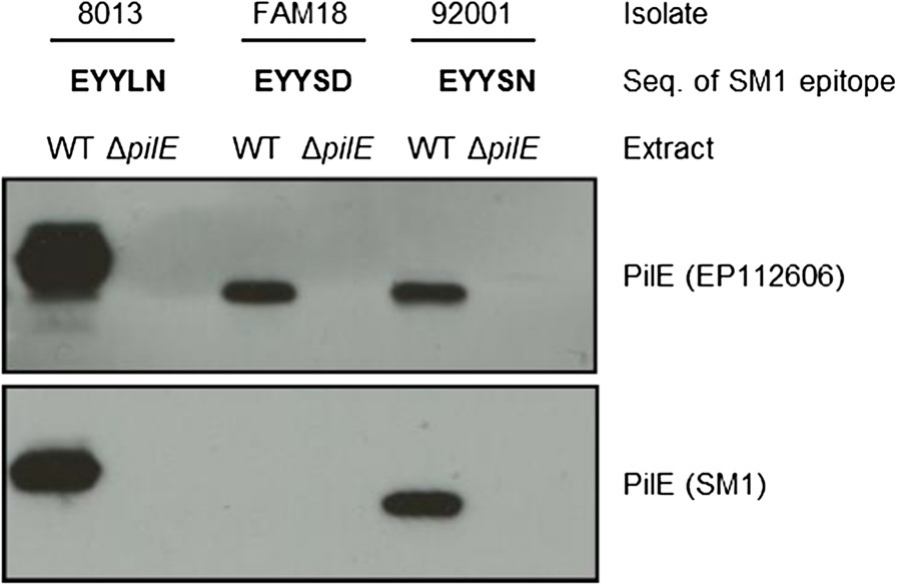
**Western blot analysis of SM1 cross reactivity with class I and class II meningococcal pilins.** Whole cell extracts from strains with class I or class II *pilE* genes encoding pilins with different SM1 epitopes, and isogenic mutants lacking *pilE* were analysed using monoclonal antibody SM1 and anti-peptide antibody EP112606. All strains express pilin, as detected by EP112606. As expected, SM1 reacts with class I pilin in 8013 but does not recognise the class II pilin from FAM18. However, pilin from isolate 92001 which has a class II *pilE* gene (allele 15) also cross reacts with SM1.

Class II pilins were initially identified by their lack of cross reaction with monoclonal antibody SM1 which was developed to differentiate between structural classes of Tfp on gonococci and meningococci [24]. SM1 recognises an epitope (EYYLN) present at the end of the conserved N-terminus of class I pilin [25]. Our analysis confirms that all the pilins encoded by the class I *pilE* contain this motif, while in the class II pilins this motif is either EYYSN (allele 15, present in 20 isolates) or EYYSD (present in all remaining isolates) (Figure 2). Western blot analysis of whole cell extracts from strains expressing class II pilin subunits with different SM1 sequences was undertaken (Figure 5). As expected, SM1 detected class I pilin in the control strain 8013 (ID 1038, allele 6, EYYLN motif) and did not recognise the class II pilin from FAM18 (ID 698, allele 3, EYYSD motif). However, class II pilin from cc5 strain 92001 (ID 597, allele 15, EYYSN motif), does cross react with SM1. This suggests that SM1 reactivity may not be sufficient to distinguish class I and class II pilin in all meningococci. We therefore compared all the pilin sequences from our analysis to examine the presence and conservation of additional differentiating features (Table 3). The sequence of the leader peptides were distinct (MNTLQKG and MKAIQKG for class I and II pilins respectively) as was the length of the C-terminus (after the second conserved cysteine), which comprises one to three amino acids in class II pilins, in contrast to seven in full-length, functional class I pilins. Most notably, all pilins encoded by the class II *pilE* genes were smaller, mostly due to a significantly shorter D-region, which is 13 or 16 amino acids in length compared with 28 to 40 amino acids in class I pilins (see Figure 2 and Table 3). Based on this analysis, we propose the adoption of a sequence-based distinction between class I and class II pilins, rather than a definition based on antigenic properties (reactivity with SM1).

**Table 3:** Defining features of meningococcal class I and class II type IV pilins

**Pilin**	**Length (AA)**	**Leader sequence**	**SM1 motif**	**Length of D-region**^**1**^
Class I	164-172	MNTLQKG	EYYLN	28-40 AA
Class II	143-152	MKAIQKG	EYYSD	13 or 16 AA
			EYYSN	

^1^Defined as the amino acids between the two C-terminal cysteine residues.

### Analysis of *pilE* in non-pathogenic *Neisseria* species

**Figure 6:**
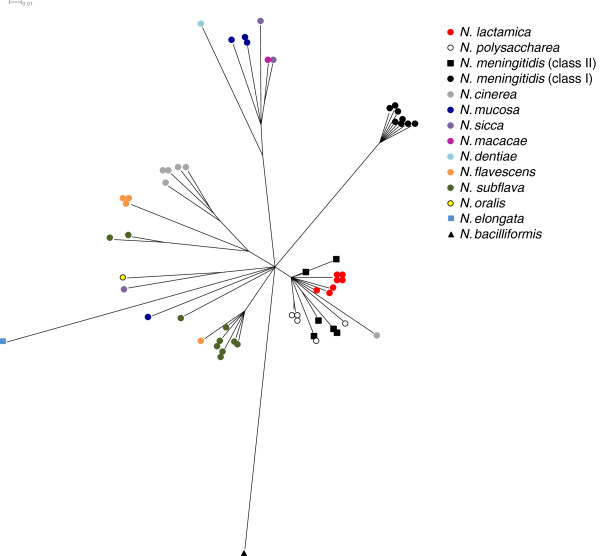
**SplitsTree decomposition network of the *pilE *gene in different *Neisseria *species.** Full-length *pilE* coding sequences were obtained from 44 representative non-pathogenic *Neisseria* genomes available in PubMLST. The *pilE* from pathogenic *Neisseria* included in this analysis were from reference strains (*i.e*. those with fully assembled, closed genomes). For comprehensive analysis we also included meningococcal class II *pilE* from isolates belonging to cc1, cc4 and cc174 obtained in this work. The species are indicated by colour code. Meningococci with class I *pilE* (black circles) form a separate branch, while the meningococcal class II *pilE* genes (black squares) are more closely related to the *pilE* from non-pathogenic *Neisseria* species, *N. polysaccharea* (white circles), *N. lactamica* (red circles) and one isolate of *N. cinerea* (grey circle).

It has previously been shown that class II *pilE* is also present in non-pathogenic *Neisseria*[27,36,38]. We therefore analysed *pilE* sequences in genomes of 46 isolates of non-pathogenic *Neisseria* available in pubMLST. This collection comprised 14 different species isolated between 1962 and 2003 from various hosts and sites. In total 44/46 WGS from non-pathogenic *Neisseria* were found to have full-length sequences homologous to *pilE* (These isolates are listed in Additional file 8). Split decomposition network analysis of these 44 *pilE* sequences with both class I and class II *pilE* genes from 14 *N. meningitidis* isolates from different clonal complexes, revealed that all meningococcal class I *pilE* clustered together (Figure 6). In contrast, the meningococcal class II *pilE* clustered on a separate branch together with *pilE* from *N. lactamica*, *N. polysaccharea* and one *N. cinerea* isolate. Other non-pathogenic *Neisseria* species displayed more divergent *pilE* sequences. We analysed the sequences flanking the putative *pilE* genes in the genomes of all non-pathogenic *Neisseria* strains where there was sufficient sequence available on the *pilE*-containing contig. Nine species contained a second putative pilin gene adjacent to the initial *pilE* homologue (see Additional file 8), in agreement with previous reports [36,38] and in these species the regions flanking *pilE* did not resemble those found adjacent to either the class I or class II meningococcal *pilE*. In contrast, the genetic organisation of open reading frames around the *pilE* gene in *N. lactamica* and *N. polysaccharea* was similar to the genomic region surrounding the meningococcal class II *pilE* gene (annotated *pilE* regions of *N. lactamica* and *N. polysaccharea* are shown in Additional file 9), although only one non-pathogenic *Neisseria* isolate (*N. polysaccharea*, isolate CCUG 27182) was found to harbour the *hp* gene which is found in the meningococcal class II *pilE* locus.

## Discussion

Two different classes of pilin (class I and class II) have been described in pathogenic *Neisseria*[24] and the genes encoding these pilins are distinguishable [29,39]. Here we have taken advantage of available whole genome sequences to provide a more comprehensive view of the distribution and genetic context of meningococcal class I and class II *pilE*. These analyses reveal a possible basis for the evident lack of gene conversion of class II *pilE* and we provide insights into the likely origin of the meningococcal class II *pilE* gene.

Previous analysis of *pilE* sequences in *N. meningitidis* isolate collections from the UK [29] and China [30] suggested an association of the class II *pilE* gene with certain lineages, especially those responsible for epidemic meningococcal disease (*i.e.* cc1, cc5 and cc11). In the present analysis, two large and well characterised isolate collections enabled the assessment of class II *pilE* distribution in isolates from a single country in one year, and in isolates collected over decades from diverse global locations. The data demonstrate that the association of class II *pilE* with these hyperinvasive lineages is maintained, regardless of the country or the year they were isolated, and highlights that class II *pilE* is present in lineages which are responsible for a large proportion of disease. For example, the 107 isolate MLST collection analysed in this study contains cc1 and cc5 isolates from countries of the African meningitis belt (ID 128 and ID 34, Additional file 3). The majority of epidemic meningococcal disease has occurred in this region and has been mostly caused by serogroup A meningococci belonging to cc1 or cc5 [40,41]; in particular three clones (ST-5, ST-7 and ST-2859, which are all part of cc5) have been largely responsible for meningococcal disease in Africa over the last 20 years [42-44].

In addition, we found class II *pilE* genes in strains from cc174 and cc4. An increase in carriage of cc174 strains was reported in the UK from 1999-2001 [45] and isolates from cc174 accounted for approximately 20% of serogroup Y meningococcal disease during 2007-2009 in the UK and were associated with disease in older age groups [46]; however, only 9/514 disease isolates from the UK in 2010-2011 belong to this complex. Therefore, there is currently insufficient information to determine whether strains belonging to this clonal complex are hyperinvasive and/or associated with epidemic disease. It is possible that the groups of strains with class II *pilE* may expand with increasing availability of strain collections and whole genome sequences and consequently the association of class II *pilE* with strains that have particular epidemiological characteristics should become clearer.

A class II pilin gene was identified in one isolate from cc4, which was of particular interest as we also identified isolates in this clonal complex that possess a class I *pilE* gene (see Additional file 10). This was inconsistent with our previous analysis which indicated that isolates within clonal complexes were uniform with respect to pilin class (*i.e*. they all have either class I or class II *pilE*). cc4 meningococci are reported to be genetically homogeneous and were responsible for epidemics in the meningitis belt from the 1960s to the early 1990s [47]. The cc4 isolate with class II *pilE* was the oldest strain in the collection, isolated in 1937, while the two strains with class I *pilE* were from 1963 and 1983, suggesting that there may have been a shift from class II *pilE* to class I *pilE* in cc4. It is interesting to note that the cc4 complex has been replaced as the major cause of disease in the meningitis belt by cc1 or more recently cc5 meningococci, both of which have class II *pilE*[47].

Our analysis confirms that class II pilins in meningococci are antigenically stable. For example, we found that a serogroup W isolate (ID 20449, M11_240427, cc11) responsible for disease in the UK in 2011, has an identical *pilE* sequence to a serogroup B isolate (ID 349, 38VI, cc11), isolated from the USA in 1964. This contrasts with the finding that every class I pilin had a different sequence, even from strains with the same sequence type, isolated from the same country, during the same year. The extensive variation of class I *pilE* is consistent with the ability of single strains to generate pilin diversity through the recombination between *pilS* cassettes and the *pilE* expression locus [48]. Indeed, for the majority of isolates with class I *pilE* the putative donor *pilS* cassettes could be identified. Although *pilS* cassettes were found in all isolates which harboured a class II *pilE* gene, it was not possible to identify any class II *pilE* sequences that contained D-region information corresponding to *pilS* cassettes. Therefore, the WGS analysis provides further evidence for a lack of *pilE*/*S* recombination in class II pilin producing meningococci. A possible explanation for the low variability of the class II *pilE* genes is the absence of the G4 sequence that is required for pilin conversion. The current hypothesis is that the formation of a guanine quartet on the lagging strand stalls DNA replication, leading to a nick on the leading strand and initiating antigenic variation [49]. Therefore, in the absence of a functional G4 adjacent to *pilE*, these strains would lack the ability to initiate pilin conversion, although recent work suggests that additional factors may contribute to the lack of pilin variation [28]. In this respect, isolates expressing class II pilin provide a useful tool for systematically dissecting the molecular and genetic elements required for pilin variation.

These results also raise questions concerning the origin of the meningococcal class II *pilE* locus. The analysis of *pilE* genes in pathogenic and non-pathogenic *Neisseria* indicates that the *N. meningitidis* class II *pilE* is more closely related to *pilE* from non-pathogenic species than to the class I *pilE* from meningococci, in agreement with previous reports [27,36]. These observations are consistent with horizontal gene transfer among *Neisseria* species which has been observed by others [38,50] and is likely, given that diverse species share a biological niche within the human host. Furthermore, we found one isolate of *N. polysaccharea* with the *hp* gene adjacent to *pilE*. This gene, which encodes a putative transposase from *Haemophilus influenzae*, is consistently found next to the class II *pilE* gene in meningococci. Therefore this provides additional evidence in support of genetic exchange around the *pilE* locus among these species.

In addition to differences in *pilE*, meningococci producing class II pilin had fewer *pilS* cassettes (1-4 *pilS*) in the region between *lpxC* and *fkbp* than isolates with class I pilin (4-11 *pilS*). One possible explanation for this finding is that the *lpxC*-*fkbp* region of existing class II strains represents a remnant of an ancestral class I *pilE*/*pilS* region, and that these class II isolates have at some point acquired a class II *pilE* gene and lost the class I expression locus and some *pilS* cassettes. Interestingly, no genomes with both a class I and a class II *pilE* gene were found in this study. It is also noteworthy that recent genomic analysis indicates that pathogenic *Neisseria* evolved from a common ancestor shared with *N. polysaccharea*[50], which has a class II *pilE* gene. This could suggest a second possible scenario in which the class II *pilE* locus is ancestral in *N. meningitidis* and the class I *pilS*/*pilE* locus is a more recent trait which has arisen in specific lineages.

Tfp are surface structures which are exposed to the host environment in both colonisation and disease, and are thus presumed targets of the immune system. Consequently pilin variation has largely been assumed to provide meningococci with an effective mechanism of immune evasion. Indeed, anti-pilus antibodies can be found in patients infected with *N. meningitidis*[51] and gonococcal Tfp containing class I pilin are immunogenic in human volunteer studies [52,53] although there is no clear role for anti-PilE antibodies in protection against meningococcal infection [54]. Our finding that some clonal complexes express conserved class II pilin subunits is not consistent with host immunity-driven pilin variation in these strains, and is reminiscent of the lack of diversification of other surface proteins such as PorB, PorA, and FetA in some hyperinvasive strains [55,56]. However, we found some evidence of class II *pilE* gene exchange between unrelated isolates (*i.e.* different clonal complexes), indicating that recombination following uptake of exogenous DNA is a potential mechanism of generating class II pilin variation. A better understanding of the immune responses to class I and class II pilins should provide insight as to why distinct hyperinvasive lineages have evolved different potential for sequence variation of the Tfp major subunit.

## Conclusions

In conclusion, we show that class II pilins can be defined by amino acid sequence and genomic context and are present in *N. meningitidis* strains which have persisted and spread globally over several decades and are responsible for meningococcal disease worldwide. Furthermore the class II *pilE* gene is present in non-pathogenic *Neisseria*, including *N. polysaccharea* and *N. lactamica*. Despite the presence of *pilS* cassettes as potential donors of genetic variation in meningococci producing class II pilins, we found no evidence that class II *pilE* recombines with *pilS* to generate pilin variation. Given the importance of Tfp in pathogen-host interaction, we propose that possession of class I or class II *pilE* is likely to impact bacterial behaviour, either through different capacity of strains to alter the sequence of the major pilus component or directly, through the inherent properties of the specific pilin subunits. For example, particular sequences of class I pilins affect key Tfp functions by influencing cell tropism or adhesion [20,57,58]. Additionally, specific amino acids in the D-region can affect pilus bundling [59] and this has recently been linked to serum resistance [23]. It is possible that class II pilins are highly conserved in certain lineages because they confer specific properties important for interactions within the host. Future work will be directed at understanding how these different pilin genes have evolved and their impact upon meningococcal biology.

## Methods

### Genome database and collections

The genome sequences of *Neisseria* strains used in this work are publically available online in the PubMLST *Neisseria* BIGSdb database (http://pubmlst.org/neisseria/). This database was developed by Keith Jolley and is sited at the University of Oxford [4]. The MRF-MGL (http://www.meningitis.org/research/genome) is an online open access library developed collaboratively by the Health Protection Agency, the Wellcome Trust Sanger Institute and the University of Oxford and is also hosted on the PubMLST website. The sequences analysed in this work were, for the most part, draft whole genome sequences and the majority were not fully assembled or closed genomes. The number and length of contigs for genomes can be found by using the “sequence bin” analysis tool on the PubMLST website. The 107 isolate MLST collection was accessed via the “Search *Neisseria* PubMLST database” web page using the “Publication” filter and selecting “Maiden et al. [2], Proc Nat Acad Sci USA 95:3140-5” from the drop down menu. The genomes of 514 meningococcal disease isolates from the epidemiological year 2010/11 in England, Wales and Northern Ireland were available in the MRF genome library (at time of writing). The genomes of 46 other *Neisseria* species were selected by excluding species names containing *meningitidis* and *gonorrhoeae*, and filtering for isolates with whole genome sequences in the drop down project menu. Each isolate has a unique ID number. Fully assembled (closed) genome sequences for reference strains MC58 (ID 240) [32], FAM18 (ID 698) [34], WUE2594 (ID 19260) [35], G2136 (ID638) [60], α14 (ID 30) [61], α710 (ID 19261) [62], M01-240355 (ID 19265) [60] , NZ-05/33 (ID 19263) [60], H44/76 (ID 237) [63], 8013 (ID 1038) [64] and *N. lactamica* 020-06 [65] are described in previous publications and are available on PubMLST.

### Identification of class I and class II *pilE* genes in meningococcal genomes

Homologs of *pilE* were identified in the selected genomes using the BLASTn online analysis tool in PubMLST. For initial searches, the default parameters (word size 11 and 1 hit per isolate) were used. The query sequence consisted of either the full-length coding sequence of previously identified class I or class II *pilE* genes (*e.g*. MC58 [32] [Genbank: NC_003112] and FAM18 [26], [Genbank: NC_008767]) respectively or unpublished class II *pilE* sequences - R. Exley). The percentage identity between the nucleotide sequence of *pilE* from MC58 (class I) and FAM18 (class II) is 69% (Clustal W), and based on previous work [29] class II *pilE* genes are significantly shorter than class I *pilE* genes (approx. 450 bp compared with ≥ 500 bp) therefore in most instances using a class I or a class II *pilE* coding sequence was sufficiently stringent to distinguish either a class I or a class II *pilE* homolog. However, to verify our findings, more comprehensive searches were also performed where the query sequence comprised nucleotides 1-180 of the *pilE* coding sequence from serogroup B strain MC58, which corresponds to the highly conserved N-terminal alpha helical region of all meningococcal Type IVa pilins [66]. Hits with >80% sequence identity over an alignment length of ≥50% of the query sequence were extracted with flanking sequence and manually inspected to identify *pilE* coding sequences and classify them as class I or class II based on the known characteristics of class I or class II pilins *e.g*: size, presence of the SM1 epitope EYYLN for class I pilins [25,26]. Using these search parameters we identified some genomes where *pilE* was not detected (Additional file 1). Given that the majority of the genome sequences are represented on contigs but are not closed, it was not possible to determine whether this was due to absence of *pilE* or insufficient contig assembly, therefore, these were excluded from further analysis. Additionally we excluded *pilE* sequences that were truncated by contig length as it was impossible to ascertain whether these were class I or class II *pilE* in the absence of full-length coding sequence. Finally, we also identified *pilE* genes that were either elongated, truncated by frameshift or had deletions compared to full-length *pilE* coding sequences. These sequences most likely resulted from gene conversion and would likely lead to production of a non-functional pilin subunit [67] and thus were also not included in further sequence analysis in this study. *pilE* was designated as NEIS0210 in PubMLST and each new nucleotide allele identified was assigned a unique number in the PubMLST definitions database. To identify whether any genomes contained more than one *pilE* gene, BLAST searches were performed using class I and class II *pilE* as query sequences, as well as selecting for up to 10 hits per isolate.

### Identification of *pilE* genes in non-pathogenic *Neisseria*

Homologs of *pilE* were identified in selected non-pathogenic *Neisseria* genomes using the BLASTn online analysis tool in PubMLST. For initial searches, the default parameters (word size 11, 1 hit per isolate and 5000 bp flanking length) were used. The query sequence consisted of the full-length *pilE* coding sequence of *N. lactamica* 020-06. Sequences were extracted and manually inspected to identify *pilE*. In species where two putative pilin genes were identified in tandem on the same contig, the gene with the higher homology to the *N. lactamica* 020-06 *pilE* was chosen for split decomposition network analysis.

### Identification of *pilS* cassettes in isolates containing class I *pilE*

We analysed pilin loci in a subset of cc41/44, cc32, and cc269 isolates in order to determine the number and diversity of *pilS* cassettes residing in each *pilE/S* locus. We initially extracted the *pilE/S* locus from each strain by performing BLASTn searches in PubMLST using *pilE* as the query sequence; all search conditions were at default setting with the exception of a designated flanking length of 10,000 bp. The *pilE*-containing contigs were copied to serial cloner files and *fkbp* and *lpxC* genes were identified based on homology to previously annotated or published sequences. Sequences where contig length was insufficient to identify flanking genes were excluded from further analysis. We next identified individual *pilS* cassettes within the *fkbp-lpxC* regions of each isolate by utilizing the Align Two Sequences tool available at NCBI to interrogate the extracted *fkbp-lpxC* region using the *pilS1* gene from *N. meningitidis* strain M01-240149 as a query sequence in tBLASTn searches performed under default conditions. Each hit with >60% sequence identity was inspected manually for features characteristic of known neisserial *pilS* cassettes, which have been defined as partial genes with homology to pilin-encoding sequences [31,68]. We delineated the 5′ and 3′ boundaries of each *pilS* cassette based on this criterion, although in some cases open reading frames extended beyond the region of pilin homology.

### Identification of *pilS* cassettes in class II *pilE*-containing isolates

The genome sequence of each isolate with class II *pilE* was first examined for *pilS*-containing regions using the BLASTn online analysis tool on PubMLST using *pilS1* and *pilS2* genes from *N. meningitidis* strain FAM18 as query sequences under default conditions. Sequence regions containing hits were extracted with up to 5000 bp of flanking sequence as allowable by contig length. We examined each extracted *pilS*-containing region for *fkbp* homology, *lpxC* homology, and number and diversity of *pilS* cassettes as indicated above. Comparisons of *pilE* and *pilS* sequences from each strain were also carried out as indicated above.

### Identification of Sma/Cla and Guanine quartet sequences

The sequence of the 66 bp Sma/Cla repeats from *N. gonorrhoeae* and *N. meningitidis* strain MC58 reported in [10,37] were used as a query sequences to scan *pilE* loci for identical sequences. Where identical sequences were not identified, we manually inspected loci for highly similar sequences. Of note, the *Sma*I (CCCGGG) and *Cla*I (ATCGAT) recognition sites were not present in the query sequence and are not consistently present flanking this repeat in all genomes. However, for consistency we have kept the name Sma/Cla repeat. The sequences reported in Cahoon and Seifert [12] were used to identify putative G4 based on sequence homology.

### Sequence analysis, annotation and illustration

The online sequence analysis tool NRDB (http://pubmlst.org/analysis/) was used to identify unique alleles and EMBOSS transeq (http://www.ebi.ac.uk/Tools/st/emboss_transeq/) was used to translate multiple coding sequences for protein sequence analysis. Nucleotide and amino acid alignments, including comparisons of *pilE* and *pilS* loci from each isolate, were carried out using the online analysis tool MUSCLE available at EBI [69] or Clustal W (http://www.ebi.ac.uk/Tools/msa/clustalw2/) [70]. Alignment was displayed using the BoxShade 3.31 program on the Mobyle platform [71]. Genome regions were annotated by performing BLAST analysis with *pilE* (class I or class II)*,* or alternatively *fkpb or lpxC* as the query sequence and downloading flanking regions (usually up to 10,000 bp) from the pubMLST website. Coding sequences on the contigs were identified based on homology to previously published and/or annotated sequences [32-34,64,72,73]. Gene diagrams of regions between *fkbp* and *lpxC* and *katA* and *prlC* by were manually constructed and drawn to scale using Microsoft PowerPoint.

### Phylogenetic network analysis

The SplitsTree decomposition network of *pilE* sequences was obtained by performing sequence alignment in MEGA 5.0 [74] and network analysis using Splitstree4 [75]. Full-length nucleotide sequences of *pilE* homologues from non-pathogenic *Neisseria* species were compared with *pilE* genes from 14 *N. meningitidis* isolates which are reference isolates with previously published and annotated whole genomes (listed in Additional files 2, 3 and 4), and/or represent lineages with class II *pilE* identified in this work (*i.e.* cc1, cc4 and cc174).

### Western blot analysis of SM1 cross reactivity of class II pilins

*N. meningitidis* strains FAM18 (ID 698), 8013 (ID 1038) and 92001 (ID 597) and isogenic mutants in which the *pilE* coding sequence is replaced by a kanamycin resistance cassette were grown overnight on Brain Heart Infusion (BHI) agar (1.5% wt/vol, Oxoid) at 37°C in the presence of 5% CO_2_. Bacteria (1 × 10^9^ CFU) were resuspended in 50 μl of sterile water and an equal volume of 2× SDS-PAGE lysis buffer was added before boiling for 10 minutes. 10 μl of each whole cell extract was separated on 12% polyacrylamide gels and transferred to PVDF membrane for Western blotting. SM1 antibody (a kind gift from M. Virji) was used at a final concentration of 1:10,000 and antibody binding was detected by incubation of membranes for one hour with a goat anti-mouse HRP conjugate (DAKO) at 1:10,000. An anti-peptide antibody generated by immunisation of rabbits with peptide CGQKSAVTEYYLNHGE (Eurogentec) was used to detect both class I and class II pilin expression at a final concentration of 1:5000, followed by an anti-rabbit HRP conjugate (1:10,000; Santa Cruz). Cross reaction was detected using ECL detection reagent (GE Healthcare).

## Availability of supporting data

The draft or complete genomes available in this article are publically available online as described. Additional files with supporting results are included with this article.

## Competing interests

The authors declare that they have no competing interests.

## Authors’ contributions

RE and CT conceived the idea for this article. MW and RE analysed the *pilE* sequences, CH and EA analysed *pilS* loci, MW and JB analysed *pilE* sequences from non-pathogenic *Neisseria*, KJ and MM devised the PubMLST database and contributed to all aspects of data extraction and analysis. RE, EA, CT, MW wrote the manuscript. All authors read and approved the final submission.

## Supplementary Material

Additional file 1 **Breakdown of *pilE *analysis of genomes analysed in this study.** Table and description of the identification of *pilE* genes in WGS analysed in this study. Click here for file

Additional file 2 ***N. meningitidis *isolates with full length class I *pilE*.** Table showing the ID, isolate name, year and country of isolation, sequence type and clonal complex information for all isolates with full length class I *pilE* identified in this work. The *pilE* allele ID is also shown. Click here for file

Additional file 3 ***N. meningitidis *isolates with full length class II *pilE*.** Table showing the ID, isolate name, year and country of isolation, sequence type and clonal complex information for all isolates with full length class II *pilE* identified in this work. The *pilE* allele ID is also shown. Click here for file

Additional file 4 ***pilE* allele in eight additional reference *N. meningitidis *isolates.** Table showing the ID, isolate name, year and country of isolation, sequence type and clonal complex information for eight meningococcal isolates. These WGS are used as references as they are published as fully assembled, closed genomes. The *pilE* allele ID is also shown. Click here for file

Additional file 5 **Schematic diagrams of class I *pilE *regions.** Schematic representation of class I *pilE/S* regions and *katA*-*prlC* regions from isolates belonging to clonal complex (cc) 41/44 and cc262. Click here for file

Additional file 6 **Schematic diagrams of class II *pilE * regions.** Schematic representation of class II *pilE* regions and *pilS* regions from isolates belonging to cc1, cc8, cc174 and cc11. Click here for file

Additional file 7 **Sequences of putative G4 and Sma Cla sequences annotated in this study.** Table of the G4 and Sma/Cla sequences identified in the *pilE*/*pilS* regions of WGS annotated in this study. Click here for file

Additional file 8 **Non-pathogenic Neisseria isolates examined in this study.** Table describing the origin and details of the non-pathogenic *Neisseria* WGS analysed in this work. The number of putative *pilE* genes identified is also indicated. Click here for file

Additional file 9 **Schematic diagrams of *pilE *regions of *N. lactamica *and *N. polysaccharea.*** Schematic representation of *pilE* regions in *N. lactamica* and *N. polysaccharea* WGS analysed in this study. Click here for file

Additional file 10 **Schematic diagram of *pilE *regions in cc4 isolates.** Schematic representation showing the genetic context of the class I or class II *pilE* genes identified in cc4 meningococcal isolates. Click here for file
