# Supplementary material for: Sequence, distribution and chromosomal context of class I and class II pilin genes of Neisseria meningitidis identified in whole genome sequences
Source: BMC Genomics. 2014 Apr 1;15:253. doi: 10.1186/1471-2164-15-253 (PMC4023411; doi:10.1186/1471-2164-15-253)
Supplement: Additional file 1 — Breakdown of pilE analysis of genomes analysed in this study. Table and description of the identification of pilE genes in WGS analysed in this study. [file 1471-2164-15-253-S1.pdf]

Additional file 1. Breakdown of *pilE* analysis in MRF-MGL and MLST collections.

	MRF-MGL	MLST
**Total no. of genomes**	514	107
**No. of genomes with *pilE* not identified**	142	19
**No. of genomes with full length *pilE* CDS**	232	56
**No. of genomes with *pilE* truncated by contig**	91	30
**No. of genomes with atypical *pilE***	49[a]	2[b]
**No. of genomes with Class I *pilE***	201	12
**No. of genomes with Class II *pilE***	31	44

[a] would be class I based on leader sequence

[b] would be class II based on leader sequence

Breakdown of *pilE* analysis in MRF-MGL and MLST genome collections. We analysed a total of 621 whole genome sequences for *pilE* genes. We were unable to detect *pilE* in 161 genomes. Given that the majority of the genome sequences are represented on contigs but are not closed genomes, it was not possible to determine whether this was due to absence of *pilE* or insufficient contig assembly. Additionally we identified 121 *pilE* sequences that were truncated by contig length. We designated some *pilE* as atypical; these were either elongated, truncated by frameshift or had deletions compared to full length *pilE* coding sequences. These sequences most likely resulted from gene conversion and would lead to production of a non-functional pilin subunit.
