# Supplementary material for: Sequence, distribution and chromosomal context of class I and class II pilin genes of Neisseria meningitidis identified in whole genome sequences
Source: BMC Genomics. 2014 Apr 1;15:253. doi: 10.1186/1471-2164-15-253 (PMC4023411; doi:10.1186/1471-2164-15-253)
Supplement: Additional file 5 — Schematic diagrams of class I pilE regions. Schematic representation of class I pilE/S regions and katA-prlC regions from isolates belonging to clonal complex (cc) 41/44 and cc262. [file 1471-2164-15-253-S5.pdf]

Additional file 5. Schematic diagrams of class I *pilE*/*pilS* (A) and *katA*-*prlC* (B) genome regions from selected meningococcal isolates.

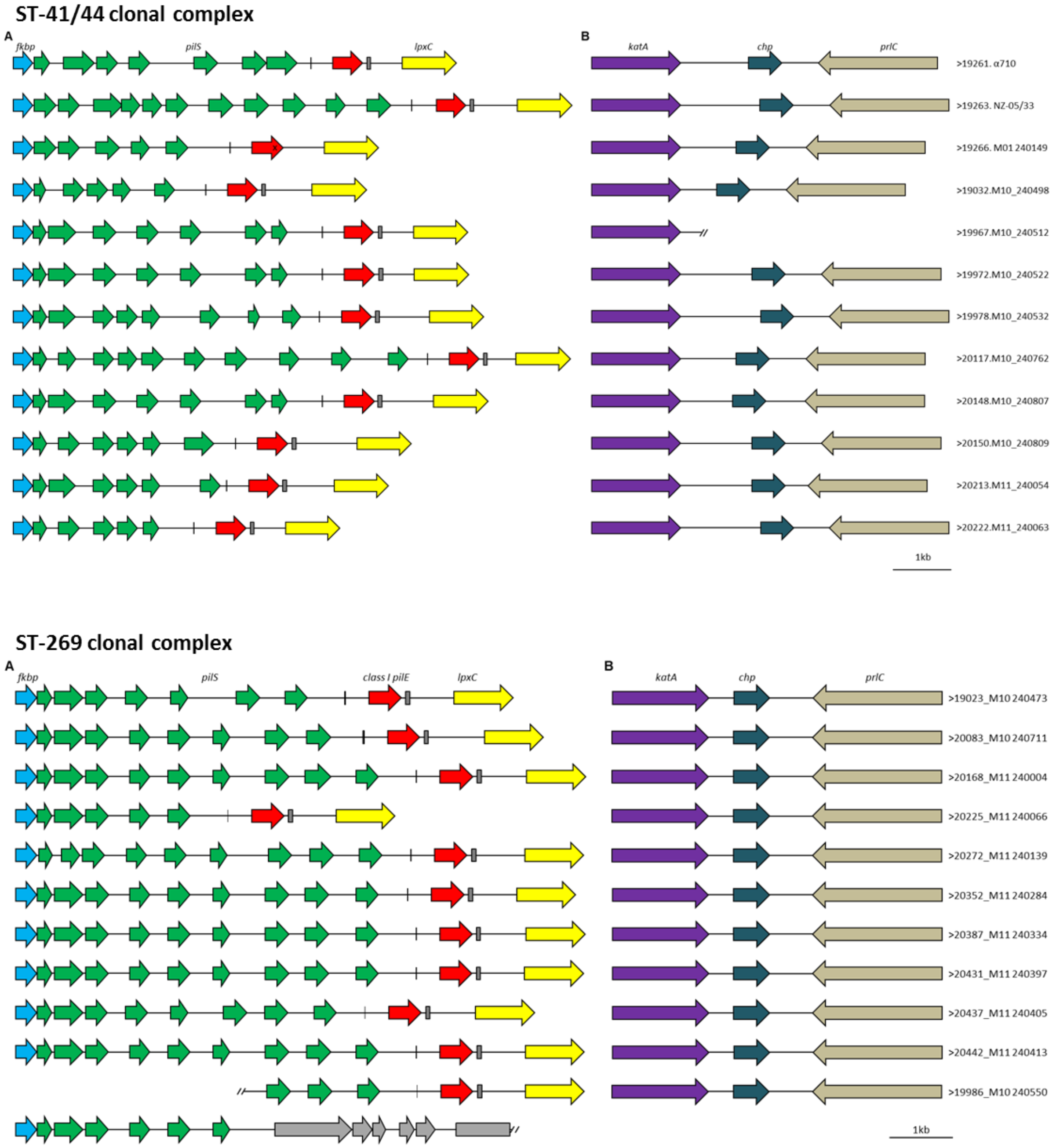

Identification code and name (>ID.name) of each isolate are indicated. Diagonal lines represent the end of the contig. X indicates the gene encodes an atypical pilin subunit which

is unlikely to result in production of functional Tfp. Putative G4 and Sma/Cla sequences are shown as black lines and hatched boxes, respectively. *lpxC*: UDP-3-O-[3-hydroxymyristoyl] N-acetylglucosamine deacetylase), *fkbp*: peptidyl-prolyl cis-trans isomerase, *pilS* : silent *pilS* cassettes, *pilE*: gene encoding major pilin subunit, *katA*: catalase, *chp*: conserved hypothetical protein, *prlC*: putative oligopeptidase A. Grey arrows represent genes with homology to the *tspB* region from FAM18, which is found in the *pilS* region of isolate 19986. Scale bar represents 1kb.
