# Supplementary material for: Sequence, distribution and chromosomal context of class I and class II pilin genes of Neisseria meningitidis identified in whole genome sequences
Source: BMC Genomics. 2014 Apr 1;15:253. doi: 10.1186/1471-2164-15-253 (PMC4023411; doi:10.1186/1471-2164-15-253)
Supplement: Additional file 6 — Schematic diagrams of class II pilE regions. Schematic representation of class II pilE regions and pilS regions from isolates belonging to cc1, cc8, cc174 and cc11. [file 1471-2164-15-253-S6.pdf]

Additional file 6. Schematic diagrams of class II *pilS* (A) and (B) *pilE* genome regions from meningococcal isolates in the pubMLST database.

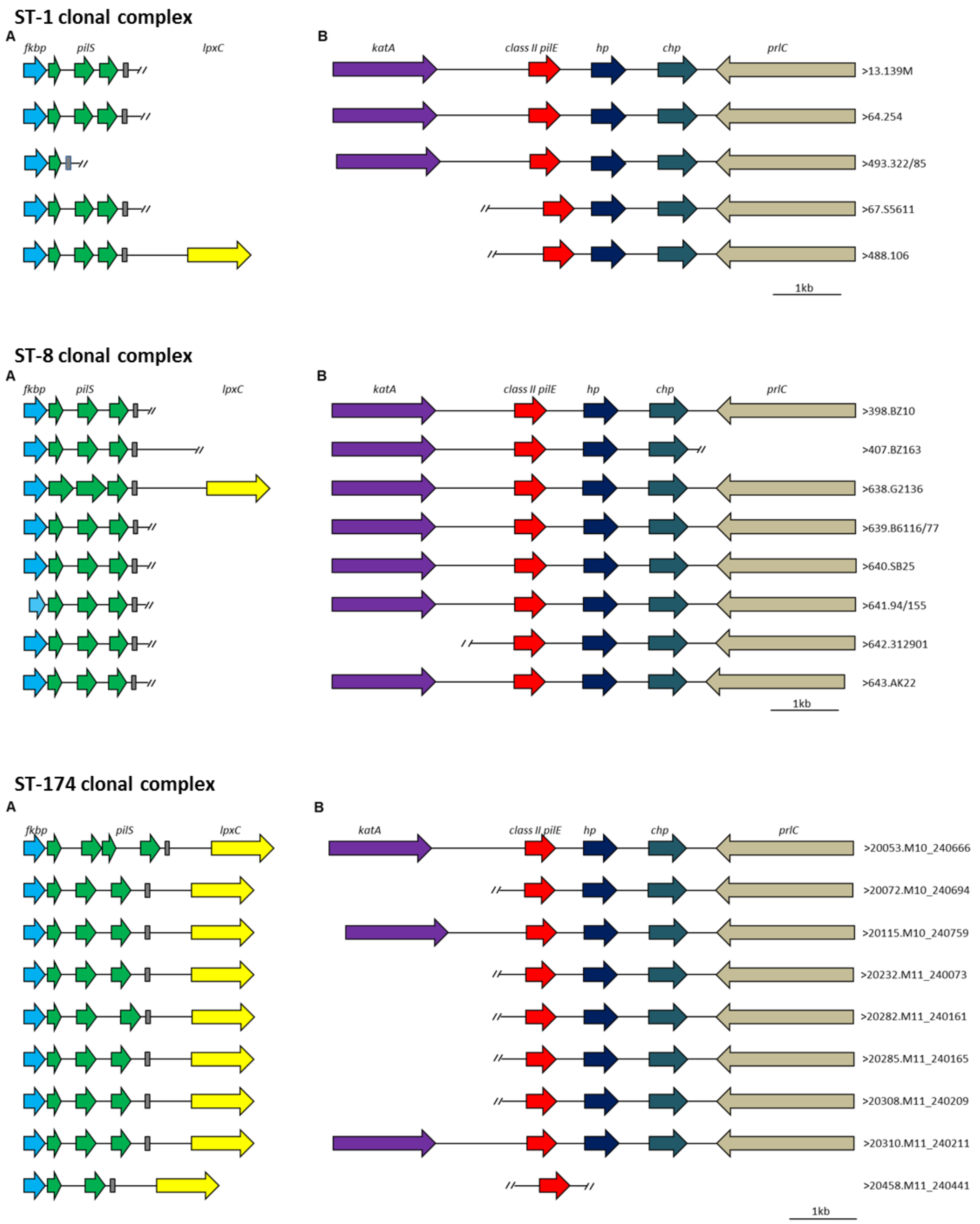

ST-11 clonal complex

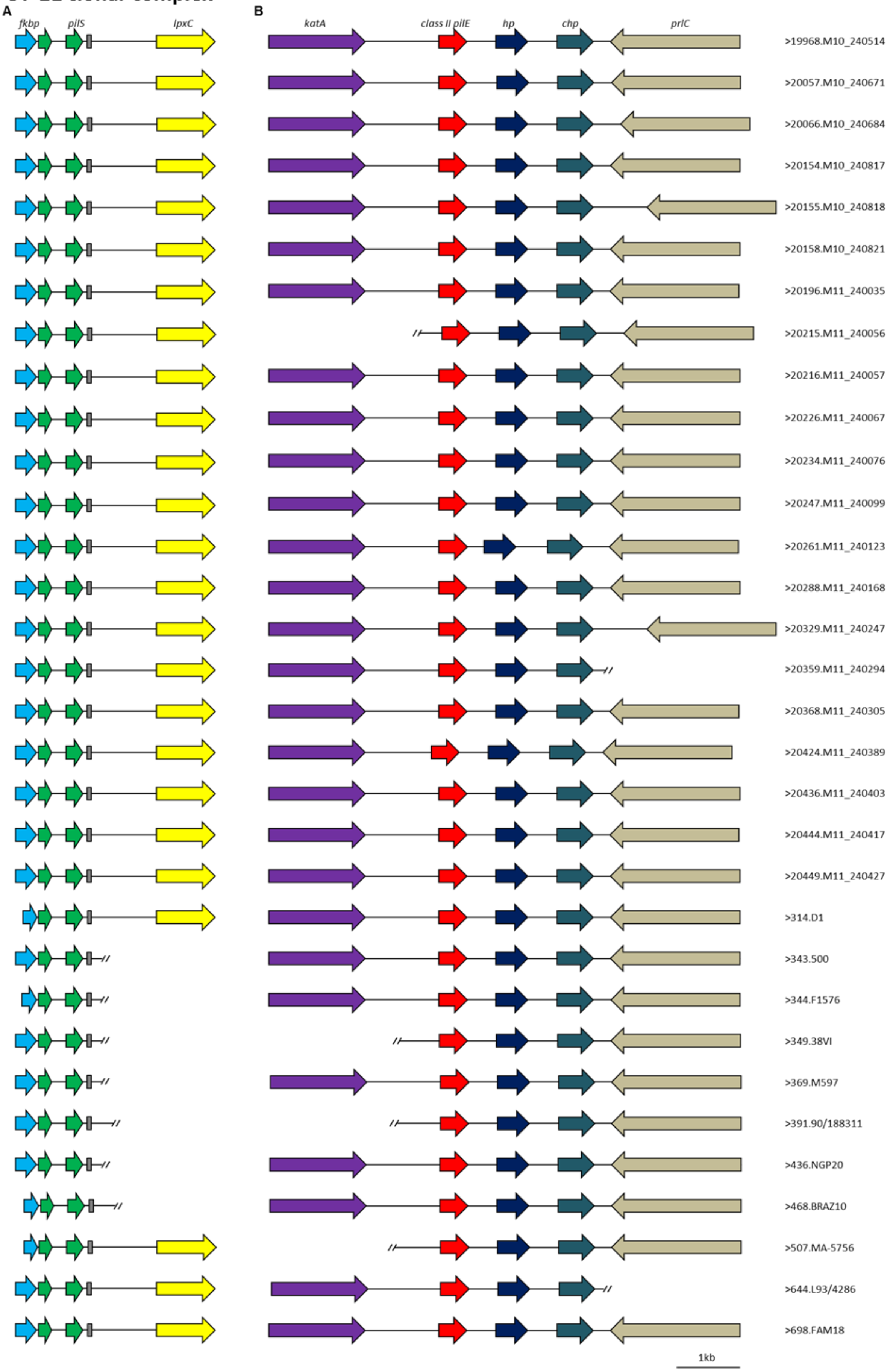

Identification code and name (>ID.name) of each isolate are indicated. Diagonal lines represent the end of the contig. Putative Sma/Cla sequences are shown as hatched boxes. *lpxC*: UDP-3-O-[3-hydroxymyristoyl] N-acetylglucosamine deacetylase), *fkbp*: peptidyl-prolyl cis-trans isomerase, *pilS* : silent *pilS* cassettes, *pilE*: gene encoding major pilin subunit, *katA*: catalase, *hp*: hypothetical protein, *chp*: conserved hypothetical protein, *prlC*: putative oligopeptidase A. Scale bar represents 1kb.
