# Supplementary material for: Sequence, distribution and chromosomal context of class I and class II pilin genes of Neisseria meningitidis identified in whole genome sequences
Source: BMC Genomics. 2014 Apr 1;15:253. doi: 10.1186/1471-2164-15-253 (PMC4023411; doi:10.1186/1471-2164-15-253)
Supplement: Additional file 9 — Schematic diagrams of pilE regions of N. lactamica and N. polysaccharea. Schematic representation of pilE regions in N. lactamica and N. polysaccharea WGS analysed in this study. [file 1471-2164-15-253-S9.pdf]

Additional file 9. Schematic diagrams of *N. polysaccharea* (A) and *N. lactamica* (B) *fkbp-lpxC* and *katA-prlC* regions.

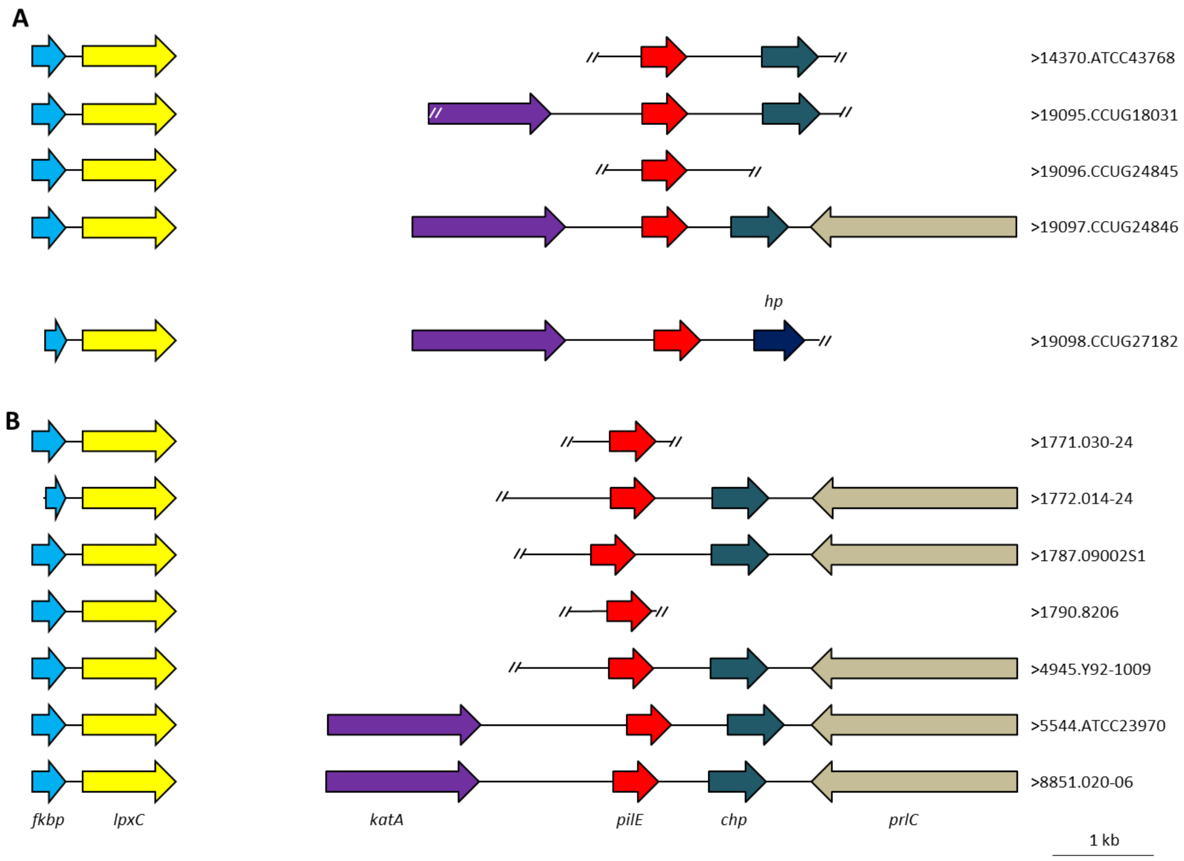

Identification code and name (>ID.name) of each isolate are indicated. Due to the draft nature of the genomes of isolates it was not possible to ascertain the genomic context of all the *pilE* genes. The end of available contiguous sequence is indicated by diagonal lines. The *N. polysaccharea* isolate CCUG27182 is the only isolate to harbour the *hp* gene. In contrast to meningococcal genomes with class II *pilE*, we did not detect *pilS* cassettes in the *fkbp-lpxC* locus in any of these isolates. *fkbp*: peptidyl-prolyl cis-trans isomerase, *lpxC*: UDP-3-O-[3-hydroxymyristoyl] N-acetylglucosamine deacetylase), *katA*: catalase, *hp*: hypothetical protein, *chp*: conserved hypothetical protein, *prlC*: putative oligopeptidase Scale bar represents 1kb.
