# Supplementary material for: Sequence, distribution and chromosomal context of class I and class II pilin genes of Neisseria meningitidis identified in whole genome sequences
Source: BMC Genomics. 2014 Apr 1;15:253. doi: 10.1186/1471-2164-15-253 (PMC4023411; doi:10.1186/1471-2164-15-253)
Supplement: Additional file 10 — Schematic diagram of pilE regions in cc4 isolates. Schematic representation showing the genetic context of the class I or class II pilE genes identified in cc4 meningococcal isolates. [file 1471-2164-15-253-S10.pdf]

Additional file 10. Schematic diagrams of class II (A) and class I (B) *pilE* genes in genomes from meningococcal isolates belonging to clonal complex 4.

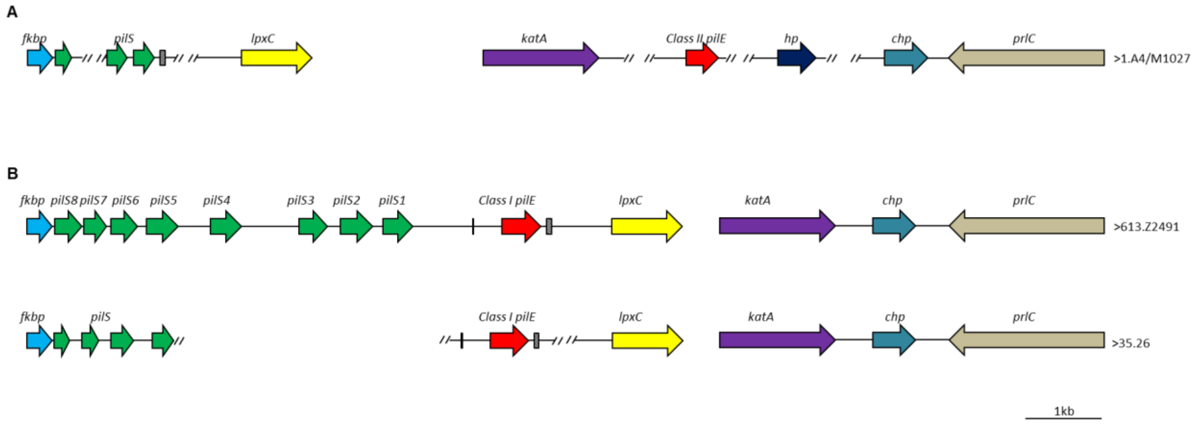

Identification code and name (>ID.name) of each isolate are indicated. Due to the draft nature of the genomes of isolates A4/M1027 and 26, it is not possible to ascertain the genomic context of the *pilE* gene and the assembly depicted is based upon the arrangement of genes in typical class I or class II loci, with the end of each contig indicated by diagonal lines. The presence of the *hp* gene in isolate A4/M1027 is consistent with all genomes harbouring a class II *pilE* allele, as is the absence of any obvious guanine quartet (G4) sequence in the region 5' to the *pilE* coding sequence. In contrast, both the G4 and Sma/Cla sequences are detected adjacent to *pilE* in isolate 26, and there is no class II *pilE* or *hp* gene in the *katA* region. Thus, we conclude that within clonal complex 4, strains can have either a class I or a class II *pilE* arrangement.

Putative G4 sequences are shown as black lines and Sma/Cla sequences are shown as hatched boxes. *lpxC*: UDP-3-O-[3-hydroxymyristoyl] N-acetylglucosamine deacetylase), *fkbp*: peptidyl-prolyl cis-trans isomerase, *pilS* : silent *pilS* cassettes, *pilE*: gene encoding major pilin

subunit, *katA*: catalase, *hp*: hypothetical protein, *chp*: conserved hypothetical protein, *prlC*: putative oligopeptidase Scale bar represents 1kb.
